# Influence of Humidity on the Elastic Modulus and Axis Compressive Strength of Concrete in a Water Environment

**DOI:** 10.3390/ma13245696

**Published:** 2020-12-14

**Authors:** Guohui Zhang, Changbing Li, Hai Wei, Mingming Wang, Zhendong Yang, Yanshuang Gu

**Affiliations:** Electrical Engineering College, Kunming University of Science and Technology, Kunming 650051, Yunnan, China; zgh_water@kust.edu.cn (G.Z.); 20192105022@stu.kust.edu.cn (C.L.); wangmingming@kust.edu.cn (M.W.); 20202105027@stu.kust.edu.cn (Z.Y.); 20202205089@stu.kust.edu.cn (Y.G.)

**Keywords:** concrete, water saturation, water environment, axial compressive strength, elastic modulus

## Abstract

Concrete structures are often in different humidity conditions that have a significant impact on the elastic modulus of concrete, therefore, systematic research on the evolution of the law of concrete elastic modulus under different humidity conditions is needed. In this study, the variation laws of the water saturation of concrete specimens with strength grades C15, C20, and C30 were obtained, and then the influence laws of the water saturation on the concrete axial compressive strength were carried out, and the prediction model of elastic modulus of concrete with respect to water saturation was constructed. The results showed that the water saturation of concrete with strength grades C15, C20, and C30 increased with an extension of immersion time, and the water saturation showed an approximately linear rapid growth within three soaking hours, reaching 47.56%, 71.63%, and 47.29%, respectively. Note, the concrete reached saturation state when the soaking time was 240 h. The axial compressive strength with strength grades C15, C20, and C30 decreased with increased water saturation, and the axial compressive strength of saturated concrete decreased by 27.25%, 21.14%, and 20.76%, respectively, as compared with the dry state concrete. The elastic modulus of concrete with strength grades C15, C20, and C30 increased with increased water saturation, and the elastic modulus of saturated concrete was 1.18, 1.19, and 1.24 times higher than those of dry concrete, respectively.

## 1. Introduction

The elastic modulus and axial compressive strength of concrete is an important mechanical parameter of concrete, which has a significant impact on the structural distortion and the expansion of a concrete track [1,2,3]. It is necessary to consider this parameter to measure and calculate the mechanical properties of concrete. The elastic modulus of concrete is mainly affected by two factors. One factor is the partial modulus of elasticity of each microscopic phase of concrete, and the other factor is the shape, size, and distribution of the internal pores and cracks [3,4,5]. The resistance to water flow under pressure is very great when the internal micropores of wet concrete are filled with water. As a component of concrete composition, water can also bear certain pressure and corresponding deformation when a compression load is applied [6,7,8,9]. The water in the pore is replaced by air due to water evaporation, since air is more compressible than the water it can be easily squeezed out or compressed when a compression load is applied, and resistance to deformation is reduced. Therefore, the influence of the internal humidity condition of concrete on the elastic modulus of concrete cannot be ignored [10,11].

There are three main viewpoints throughout the research results related to elastic modulus of wet concrete. The first viewpoint was that a wet state has no effect on the elastic modulus of concrete; the experimental results of Bjerkeli et al. [12] showed that humidity did not affect the elastic modulus of concrete. Morley et al. [13] found that both water and air existed at the same time in the pores of concrete, the water in the pores was not incompressible, and the pore water had no effect on the elastic modulus of concrete. The second viewpoint was that the elastic modulus of concrete increased with an increase in humidity. Davis et al. [14] found that the elastic modulus of saturated concrete was 12–30% higher than that of dry concrete. Johnston and Cook [15,16] found that elastic modulus was reduced by 32%, when the relative humidity of concrete was reduced from 100% to 30%. Yaman et al. [17,18] found that the Poisson’s ratio and elastic modulus of saturated concrete were higher than those of dry concrete under the same porosity. Liu et al. [19] obtained that the elastic modulus of fully saturated concrete was 30% higher than that of completely dry concrete through experiments. Wang and Li [20] believed that the elastic modulus of dry concrete was reduced by 15% with respect to saturated concrete. The study of Hou et al. [21] showed that the difference of curing humidity also caused the difference of elastic modulus. The elastic modulus of concrete cured in an environment with higher relative humidity could be increased by 15% as compared with a dry environment, and with the extension of curing age, the difference could become more significant. The third viewpoint was that the elastic modulus of concrete, at different levels of humidity, was affected differently by strain rate. Wu et al. [22] and Rossi et al. [23] found that the loading rate had a significant effect on the elastic modulus of saturated concrete. With an increase in saturation, the elastic modulus of concrete increased by 29–77% when strain rate was 10^−6^/s and increased by 30–63% when strain rate was 10^−3^/s, respectively. The study of Wang and Li [24] showed that the elastic modulus of concrete increased by 20% when the strain rate was 10^−6^/s and increased by 35.5% when the strain rate was 10^−3^/s. The results of Rossi et al. [25] showed that the elastic modulus of saturated concrete increased by 26.6% when the strain rate was 10^0.3^/s, but decreased by 4.5% when the strain rate was 10^−6^/s.

In conclusion, the physical experiment conclusion on the effect of humidity on the elastic modulus of concrete is still controversial, but the main conclusion is that the elastic modulus of concrete increases with an increase in humidity, and the larger the strain rate, the higher the increase of the elastic modulus of concrete. At present, research results still have the following three problems:

1. The elastic modulus of single strength grade concrete has been researched in previous studies, however, the sensitivity of elastic modulus affected by humidity conditions of concrete with different strength grades was different.
2. In the physical experiment of concrete elastic modulus, the research range of humidity was mostly dry and saturated humidity conditions, and there was no intermediate transient excessive humidity condition.
3. The humidity level was expressed in terms of water content in previous studies, but the water content was an absolute quantity. The proportion of pores in concrete filled with water could be expressed by the moisture content.

This research was carried out on the evolution law of elastic modulus and axial compressive strength of concrete with three different strength grades, i.e., C15, C20, and C30, in six different humidity conditions to systematically research the influence law of humidity on elastic modulus of concrete. The concrete humidity conditions included completely dry, fully saturated, partially saturated, and intermediate humidity conditions. The humidity condition was expressed in terms of water saturation, and the proportion of pores filled with water was reflected through water saturation (range 0–100%). The prediction model of concrete elastic modulus with strength grades C15, C20, and C30 to water saturation was constructed. The research results on the variation law of concrete axial compressive strength and elastic modulus under different humidity conditions provides a foundation for the evaluation of the mechanical properties of concrete structures with different levels of humidity.

## 2. Experimental Materials, Equipment, and Design

### 2.1. Experimental Materials and Equipment

Composite Portland cement with P·C 32.5R grade was used in this test. The fine aggregate was selected from well-graded natural medium sand. The fineness modulus was 2.43, the clay content was 0.8%, the silt content was 0.8%, the apparent density was 2590 kg/m^3^, and the accumulation density was 1540 kg/m^3^. The coarse aggregate was selected from pebbles, and the clay content of pebbles was 0.6%, the apparent density was 2650 kg/m^3^, the bulk density was 1563 kg/m^3^, and the maximum aggregate particle size was 40 mm. The strength grades of the concrete were C15, C20, and C30, which were cured for 28 days under standard conditions (20 ± 2 °C, RH > 95%) according to the Hydraulic Concrete Mix Design Code DL/T5330-2005 [26]. The mix proportion and main parameter of concrete are shown in Table 1. The concrete specimen was a cylindrical specimen which was 150 × 300 mm. The standard curing and mixing process were in accordance with Hydraulic Concrete Test Code SL 352-2006 [27].

**Figure 1:**
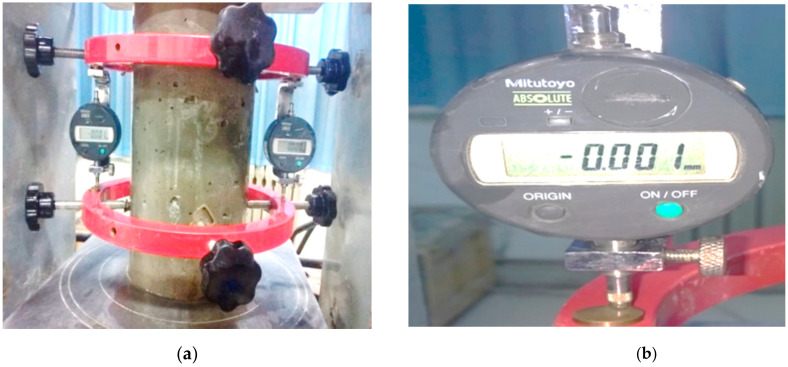
Elastic modulus tester for concrete. (**a**) Elastic modulus tester; (**b**) Electronic micrometer measured displacement.

The temperature of an electric constant temperature blast drying oven ranged from room temperature to 300 °C, its working size was 800 × 800 × 1000 mm, its temperature uniformity was ±2.5%, and its temperature fluctuation was ±1 °C. The precision of electronic scales was 1 g. The maximum test force of the microcomputer-controlled electro-hydraulic servo pressure testing machine was 2000 kN and its test force accuracy was 1%. The component composition of the elastic modulus’ tester of the concrete is shown in Figure 1a; the distance between the upper and lower rings is 150 mm, and the distance from the lower ring to the base is 75 mm. The electronic micrometer of the elastic modulus’ tester is shown in Figure 1b; the range of the electronic micrometer is 0–1 mm and its accuracy is 0.001 mm.

### 2.2. Experimental Design

The control factors of the experiment were the strength grade and the water saturation of the concrete. According to the preset factors, the concrete strength grade was set as C15, C20, and C30. The water saturation was obtained by soaking for different time periods; the immersion time periods were set as 0, 3, 10, 24, 72, and 240 h, and six humidity conditions from a completely dry state to a nearly saturated state were obtained, respectively. In this study, eighteen experimental groups were set, and six specimens were set in each experimental group, among which three specimens were used to measure the axial compressive strength of the concrete and the other three specimens were used to measure the elastic modulus of the concrete. A total of 108 specimens were tested. The specific experimental group setting is shown in Table 2.

## 3. Experimental Methods

A_0_, B_0_, and C_0_ were three completely dry groups. After standard curing for 28 days, the specimens were roasted using an electrothermal constant temperature and air-blown drying oven at 105 °C, until the vaporization mass per unit surface area was less than 0.002 kg/(h × m^2^) per unit time, which was deemed to have reached the completely dry state. The axial compressive strength and elastic modulus were measured, after natural cooling. A_1_–A_5_, B_1_–B_5_, and C_1_–C_5_ were the remaining fifteen experimental groups with different water saturations. First, the specimen was made to reach the dry state, and the dry process was the same as that of the completely dry group. After the specimen was cooled naturally, the mass $m_{0}$ of the completely dry specimen was recorded, then the specimens were put into the water tank in groups, and tap water was slowly added to make the water level flush with the top surface of the specimens to ensure that the specimens could still be submerged after water absorption. After A_1_–A_5_ were soaked for 3, 10, 24, 72, and 240 h, in turn, the specimens were taken out of the water tank and placed on an iron rack to control the water, for five minutes. Then, the surface was wiped with a wet cloth so that there was no visible water on the surface, but it still remained moist. The mass $m_{i}$ of specimens after soaking was measured. When the water absorption rate of the concrete specimens (the mass of water absorbed per unit surface area of concrete specimens per unit time) was no more than 2 × 10^−3^ kg/(m^2^ × h), the specimens were considered to have reached the approximate saturation state, and the mass of concrete specimens, in the saturated state, was $m_{w}$. When the mass was measured, the specimens were gently handled to prevent the edges and corners from breaking off, which would affect the accuracy of the quality measurement. The water saturation of the specimens was calculated according to Equation (1), and the water saturation of each experimental group was the average of the six specimens. Finally, the axial compressive strength and elastic modulus were tested, and saturation, axial compressive strength, and elastic modulus of the remaining ten experimental groups of B_1_–B_5_ and C_1_–C_5_ were determined by the same method as:

(1) $$S_{i}=\frac{m_{i}-m_{0}}{m_{w}-m_{0}}\times 100\%$$

where $s_{i}$ is the water saturation (%), $m_{i}$ is the mass of specimen under different immersion time (kg), $m_{w}$ is the mass of saturated specimen (kg), and $m_{0}$ is the mass of dry specimen (kg).

The axial compressive strength was measured using a fully automatic microcomputer servo universal testing machine. Concrete specimens were placed on the underplate of the press with geometric alignment. The loading rate was 0.3 MPa/s and the loadings were uniform and continuous. When the specimen was close to failure and began to deform rapidly, the press accelerator was stopped and adjusted, until the specimen was damaged, and then the maximum load *P* was recorded. The axial compressive strength of each experimental group was the arithmetic average of the measured values of three specimens. If the difference between any measured value and the median value exceeded 15% of the median value, the median value was taken as the measured value. If the difference between the two measured values and the median value exceeded the above provisions, the test results of the group were invalidated. The axial compressive strength, *f*_c_, was calculated according to Equation (2) as follows:

(2) fc=PA

where P is the failure load of axial compressive force (N), A is the cross-sectional area of concrete specimens (mm^2^), and $f_{c}$ is the axial compressive strength (MPa).

For the determination of elastic modulus, first, each specimen was preloaded and the load was uniformly applied to 40% of the axial compressive strength *f_c_*, which was the control load $P_{2}$ of the elastic modulus test. The loading rate was 0.3 MPa/s, and the unloading speed was the same until zero and was repeated three times, until the difference between the two adjacent deformation was no more than 0.003 mm, otherwise the above process was repeated. Notably, when the difference between the deformation values obtained on both sides of a specimen was greater than 20% of the average deformation values on both sides, the position of the specimen was adjusted until the requirements were met. After preloading, the specimens were formally tested, and the loading rate was the same as that during preloading. First, it was loaded to the initial load $P_{1}$ with the stress of 0.5 MPa, and the load was continuously loaded for 30 s, the initial deformation value was tested. Then, the load was increased, in turn, and the deformation value was measured, and the deformation value under each load (at least 6) was recorded. When the loading stress reached 50% (0.5$f_{c}$) of the axial compressive strength, the micrometer was removed. The static compressive elastic modulus $E_{c}$ of the specimen was calculated according to Equation (3) as follows:

(3) $$E_{c}=\frac{P_{2}-P_{1}}{A}\frac{L_{0}}{\Delta L}$$

where $P_{1}$ is the initial load which the stress was 0.5 MPa (N), $P_{2}$ is the control load which the stress was 0.4$f_{c}$ (N), A is the section area of the specimen (mm^2^), $L_{0}$ is the standard spacing for measurement (mm), and ΔL is the deformation value of the specimen from $P_{1}$ to $P_{2}$ (mm).

The final test result was the arithmetic average of the measured values of the three specimens for elastic modulus. If the difference between the axial compressive strength of one specimen after the elastic modulus was measured and that of the three specimens was more than 20% of the latter, the measurement result of the elastic modulus of the specimen was discarded, and the average value of the remaining two specimens was taken as the final test result.

## 4. Experimental Results and Analysis

### 4.1. Water Saturation of Concrete

**Figure 2:**
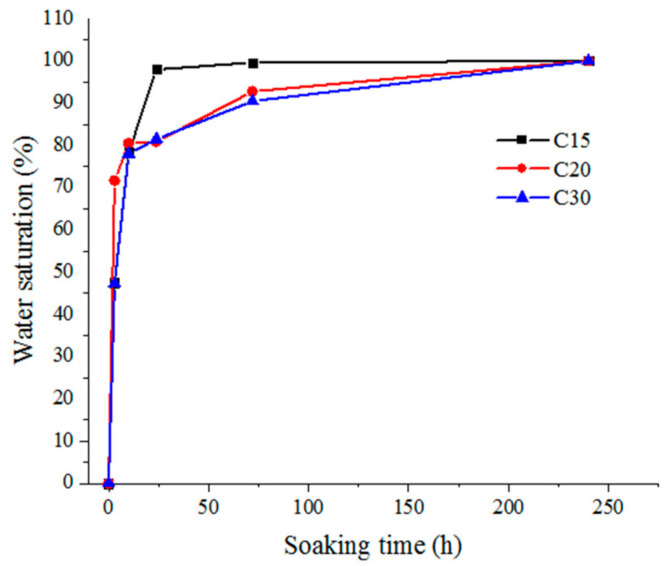
The variation law of concrete saturation with different soaking time periods.

The water saturation of each experimental group was the arithmetic mean value of the water saturation of six specimens. Figure 2 shows the variation law of water saturation of concrete cylinder specimens with strength grades C15, C20, and C30, under different immersion time periods.

It can be seen from the Figure 2 that the variation laws of the water saturation of concrete with strength grades C15, C20, and C30 under different immersion times were basically similar, and all increased with extended immersion time periods. When the immersion time was within the range of 0–3 h, the water saturation of the concrete specimens with strength grades C15, C20, and C30 increased rapidly in an approximate linear manner, reaching 47.56%, 71.63%, and 47.29%, respectively. Under the condition of the same immersion time, the lower the concrete strength grade was, the faster its water absorption rate was, and the higher its water saturation was. For example, when the immersion time was 72 h, the water saturation of concrete with strength grades C15, C20, and C30 reached 99.59%, 92.75%, and 90.48%, respectively. The water saturation of concrete with strength grade C15 was 1.11 times higher as compared with the strength grade of C30. After the immersion time reached 240 h, the concrete with strength grades C15, C20, and C30 had reached the approximate saturation state. It is worth noting that the water absorption process of concrete was complex and slow, and it could not reach the absolute saturation state in a short time, therefore, the saturation state defined in this paper was the approximate saturation state.

When the concrete had soaked for 3–24 h, at this stage, the water mainly entered into the dry concrete pores through adsorption and surface diffusion. A water adsorption layer formed on the pore wall, therefore, the coefficient formed was mainly surface water absorption. The water saturation variation rule of C15 concrete soaked for 3–24 h was not exactly consistent with C20 and C30 concrete. The water saturation variation rule of C15 concrete soaked for 24–72 h was not exactly consistent with C20 and C30 concrete, and many repeated experiments were carried out in order to confirm the authenticity of this conclusion, indicating that this experimental conclusion was not accidental. When concrete was soaked for 24–72 h, as the concrete internal humidity continued to increase, water continued to be transmitted to the specimen in the form of a water film, and the form of water absorption was seepage. At this moment, the strength of seepage was closely related to the quantity and size of the concrete pores. The porosity of C15 concrete was higher than C20 and C30 concrete, and therefore the water saturation variation rule of C15 concrete soaked for 24–72 h was not exactly consistent with C20 and C30 concrete. Research on the change law of concrete water absorption rate influenced by porosity will be completed in future research work.

### 4.2. Axial Compressive Strength of Concrete

**Figure 3:**
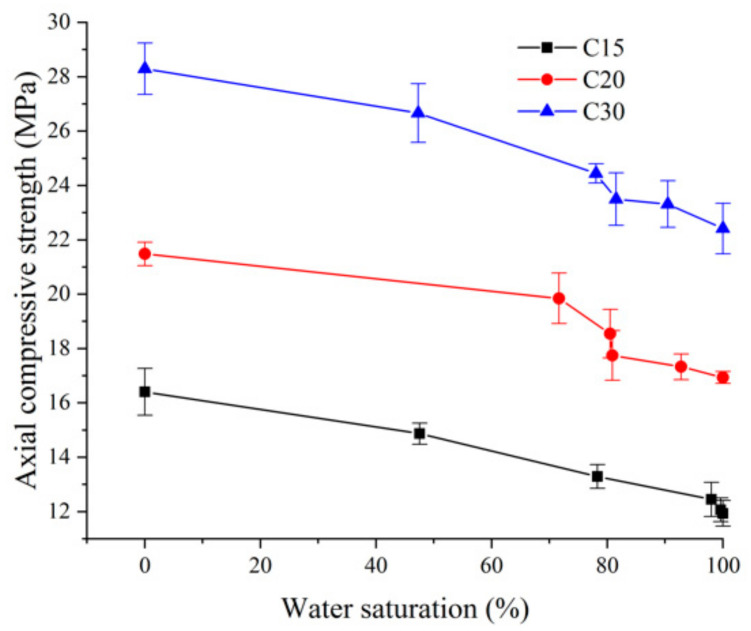
The axial compressive strength with different saturations (S.D. as error).

In order to determine the compression load, *P_2_*, in the process of elasticity modulus measurement, the axial compressive strength of the cylinder specimens was measured. The axial compressive strength was tested by using a microcomputer-controlled electro-hydraulic servo pressure testing machine. The loading rate was 0.3 MPa/s. The maximum test force of the microcomputer-controlled electro-hydraulic servo pressure testing machine was 2000 kN, and its test force accuracy was 1%. The entire test procedure for axial compressive strength was strictly in accordance with the People’s Republic of China Industry Standard (SL 352-2006) Test Code for Hydraulic Concrete [27]. The curve changes with error bars of axial compressive strength are shown in Figure 3. For measuring experimental error, the maximum standard deviation of the experimental data was 0.944.

**Figure 4:**
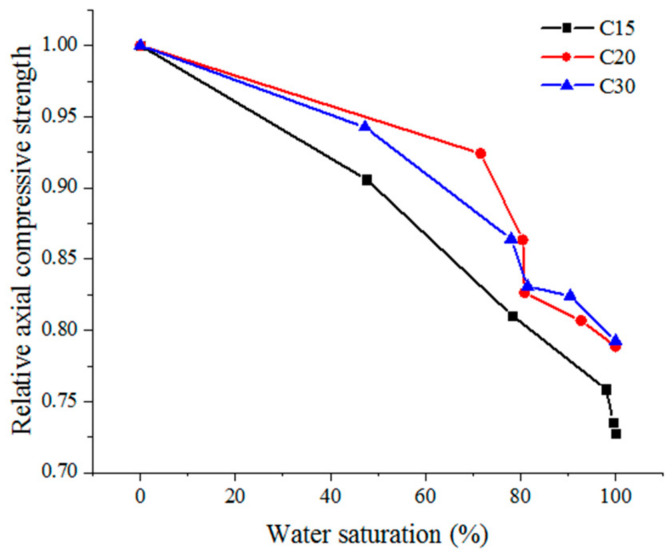
The relative axial compressive strength under different water saturation values.

The axial compressive strength of concrete under different water saturation tested values are shown in Table 3. Axial compressive strength varies under different saturation values and completely dry states are highlighted by relative axial compressive strength. Relative axial compressive strength was the ratio of axial compressive strength with different water saturation values to axial compressive strength with complete drying, therefore, relative axial compressive strength was nondimensional. Figure 4 shows the variation law of the relative axial compressive strength under different water saturation values.

It can be seen from Table 3 and Figure 4 that the axial compressive strength of concrete with strength grades C15, C20, and C30 decreased with an increase in water saturation, and the influence of water saturation was significant. For example, the axial compressive strength of the approximately saturated experimental group (A_5_, B_5_, and C_5_) decreased by 27.25%, 21.14%, and 20.76%, respectively, as compared with the completely dry experimental group (A_0_, B_0_, and C_0_). Under the same water saturation conditions, the lower the strength grade of concrete was, the greater the axial compressive strength was reduced. For example, the axial compressive strength of concrete with strength grade C15 and water saturation of 80% decreased by 19% as compared with the completely dry state, while the axial compressive strength of concrete with strength grade C30 and water saturation of 80% decreased by 14% as compared with the completely dry state, therefore, the axial compressive strength of concrete with low-strength grade was more sensitive to water saturation.

### 4.3. Elasticity Modulus of Concrete

**Figure 5:**
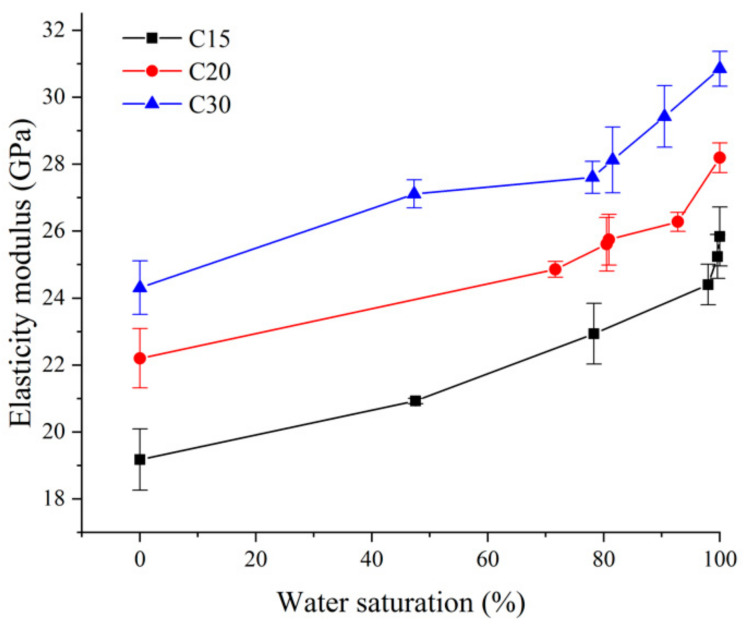
The elasticity modulus with different saturations (S.D. as error).

The elasticity modulus values under different water saturation values were tested according to the People’s Republic of China Industry Standard (SL 352-2006) Test Code for Hydraulic Concrete [27]. The test procedure of elastic modulus is described in detail in Section 3, experimental method. The curve changes with error bars of elasticity modulus are shown in Figure 5. For measuring experimental error, the maximum standard deviation of experimental data was 0.983.

The variations of elasticity modulus of concrete with strength grades C15, C20, and C30, from a completely dry state to an approximately saturated state, are shown in Table 4. It can be seen from Table 4 that the elasticity modulus of concrete with strength grades C15, C20, and C30 all showed an increasing trend with an increase in water saturation. For example, the elasticity moduli of concrete with strength grades C15, C20, and C30 in the saturated state were 1.18, 1.19, and 1.24 times higher as compared with the completely dry state, respectively. Under the same water saturation conditions, the higher the strength grade of concrete was, the greater the elasticity modulus of concrete increased. For example, when the water saturation was 48%, the elasticity modulus of concrete with strength grade C30 increased by 12% as compared with the completely dry state, while the elasticity modulus of concrete with strength grade C15 increased by only 6% as compared with the completely dry state, and this increase amplitude was 50% of the elasticity modulus of concrete with strength grade C30, therefore, the elasticity modulus of concrete with high-strength grade was more sensitive to water saturation.

According to the experimental results of concrete elasticity modulus under different water saturation values, a two-parameter prediction model of concrete elasticity modulus with respect to water saturation was established, as shown in Equation (4). A comparative analysis of the predicted value and the experimental value in this research of concrete elasticity modulus is shown in Figure 5. Curve fitting parameters of prediction Equations (4) are shown in Table 5. The predicted value by Equation (4) and tested value in reference [19] of elasticity modulus are shown in Table 6. The prediction equation is as follows:

(4) $$E_{ci}=\alpha S_{i}+\beta$$

where $E_{ci}$ is the elasticity modulus of concrete under different water saturation (GPa), $S_{i}$ is the water saturation (%), and α and β are model function constants.

**Figure 6:**
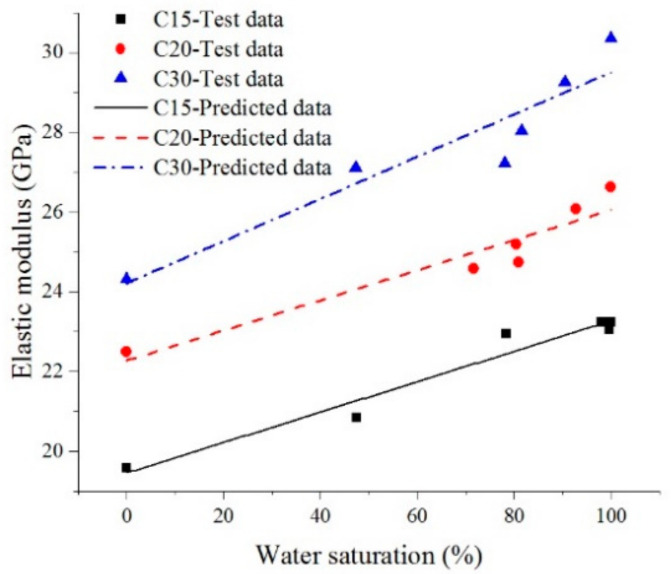
Comparative diagrams between the experimental values and fitted curves of elasticity modulus.

**Figure 7:**
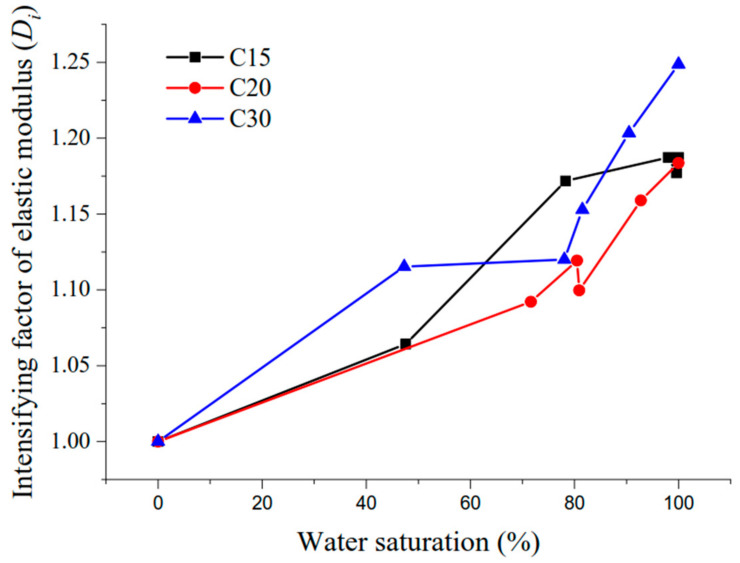
The elasticity modulus enhancement factor under different water saturation values.

It can be seen from Table 6 and Figure 6 that the test value of elasticity modulus of concrete at different water saturations was in good agreement with the predicted value of elasticity modulus of concrete. The maximum relative error of predicted value by Equation (4) and tested value reference [19] was −9.23%, because the concrete mixture ratio and experimental conditions in reference [19] were different from those in this research, and the specific parameters in the prediction model were different because Liu et al. [19] used moisture content as the measurement index of humidity and the humidity variation range was different. The prediction model (Equation (4)) had a good correlation, which could be used to predict the elasticity modulus of concrete at different water saturation values. In order to further explore the influence law of water saturation on the concrete elasticity modulus, the enhancement factor of the concrete elasticity modulus affected by water saturation is defined as *Di*, and the enhancement factors of the concrete elasticity modulus with strength grades C15, C20, and C30 affected by water saturation are defined, in accordance with Equation (5) (Figure 7 shows the variation curve of the elasticity modulus enhancement factor of concrete under different water saturation values) as follows:

(5) $$D_{i}=\frac{E_{i}-E_{o}}{E_{o}}$$

where $D_{i}$ is the elasticity modulus enhancement factor, $E_{o}$ was the elasticity modulus with completely dry state (GPa), and $E_{i}$ is the elasticity modulus under different water saturation (GPa).

As shown in Figure 7, the elasticity modulus enhancement factor tended to increase with an increase in water saturation. For example, the elasticity modulus enhancement factor of concrete with strength grades C15, C20, and C30 under water saturation of 80% were 1.06, 1.05, and 1.05 times higher relative to the saturation of 48%. Under the same water saturation condition, the higher strength grade of the concrete was, the higher the elasticity modulus enhancement factor was, therefore the elasticity modulus of concrete with a high strength grade was more sensitive to the water saturation, but within a range of 50% to 80% water saturation, there was a degree of dispersion.

## 5. Discussion

The water in concrete was split into physically free water and chemically combined water according to the existing form [28]. The changing mechanism of the elasticity modulus under saturated concrete was revealed according to the different types water on the concrete elasticity modulus.

**Figure 8:**
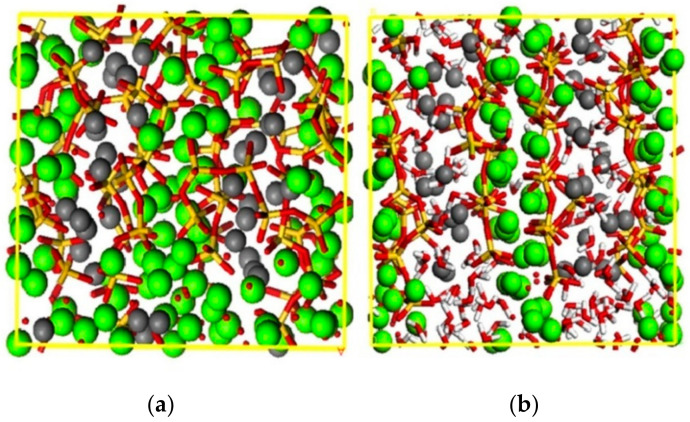
Snapshots of molecular structures of C-S-H gel samples from the dry state to the saturated. The yellow and red chain structures are Si–O_s_, the gray spherical structure are calcium atoms Ca_w_ in the interlayer region, the green spherical structure are calcium atoms Ca_s_ in the layered structure, and the red and white chain structures are water molecules. (**a**) Dry state; (**b**) Saturated state.

1. Physically free water A large number of pores and microcracks existed in the concrete, and the concrete elasticity modulus was weakened. When the concrete structure was in a nonpressurized water environment, the water entered the active pores of the concrete under the action of capillary adsorption force, and the active pores were filled by free water. Since the volume modulus of water was different from the matrix, the free water in the active pores of concrete impeded the deformation of the matrix, and the rigidity of the pores and microcracks were enhanced in the compressive elastic deformation stage under the external load. In addition, it can be seen from microhydrodynamics that the surface tension and viscous force of the water in the pores were both large, which contributed to the shear modulus of concrete in the elastic stage and ultimately the elasticity modulus of saturated concrete was enhanced. For completely dry concrete, pores and microcracks were filled with air and the elasticity modulus of air was very small, therefore the elasticity modulus of dry concrete was lower than the saturated concrete.
2. Chemically combined water The main hydration product of cement paste was C-S-H, which accounted for 60–70% of the total volume of hydration product, and its deformation characteristics directly influenced the macroscopic deformation characteristics of concrete. According to Hou et al. [29], the molecular structure diagram of C-S-H in the dry and saturated state are shown in Figure 8. The influence of the number of water molecules on the deformation of C-S-H was analyzed based on the perspective of microscopic molecular structure.

The molecular structure of C-S-H is one layer of calcium oxygen laminar structure sandwiched between two layers of silicon oxygen tetrahedron chain [30,31,32]. The molecular structure diagram in the completely dry state is shown in Figure 8a. As shown in Figure 8a, the oxygen atoms, O_W_, in water molecules form Ca_W_–O_W_ and Ca_S_–O_W_, and were partially replaced by O_S_ (oxygen atom in the C-S-H molecule) in Ca_W_–O_S_ and Ca_S_–O_S_. Because Ca_W_–O_S_, Ca_S_–O_S_, and O_S_–H_W_ had smaller bond lengths than Ca_W_–O_W_, Ca_S_–O_W_, and O_W_–H_W_, these chemical bonds decreased in length and increased in strength. The atoms were closely arranged and obviously disordered; the dislocation of atoms was caused during the process of compression, and the deformation capacity of the whole C-S-H molecular system was enhanced, therefore, the elastic modulus of concrete decreased. With an increase in water saturation of the concrete [29,33], as shown in Figure 8b, most of the water molecules passed into the C-S-H mezzanine area, a few scattered in the silica tetrahedron chain and the calcium oxide lamellar region, oxygen atoms O_w_ in the water molecules replaced part of the O_s_ in the Ca_w_–O_s_ and Ca_s_–O_s_ (the oxygen in the C-S-H molecule) in the formation of Ca_w_–O_w_ and Ca_s_–O_w_, and single O_s_–H_w_ turned into double layer or multilayer O_s_–H_w_ and O_w_–H_w_. The mezzanine area of the whole molecular system was increased, atoms that overlapped were significantly reduced, the distance between two atoms was increased, and it was difficult to cause atomic dislocation movement during the stage of compressive deformation. When the chemical bond breaks, the amount of deformation decreases, and the deformation capacity of the C-S-H molecular system weakens, thus, increasing the macroscopic elasticity modulus of saturated concrete [33,34,35].

## 6. Conclusions

1. The water saturation of concrete increased with an extension in immersion time. The lower the strength grade of concrete, the faster its water absorption rate and the higher the water saturation reached under the same immersion time condition.
2. The concrete axial compressive strength decreased with an increase in water saturation, and the effect of water saturation was significant. The axial compressive strengths of concrete with strength grades C15, C20, and C30, under approximate saturation, decreased by 27.2%, 21.1%, and 20.8%, respectively, as compared with the completely dry state. The concrete axial compressive strength with low-strength grade was more sensitive to the water saturation.
3. The concrete elasticity modulus increased with an increase in water saturation. The elasticity moduli of concrete with strength grades C15, C20, and C30, under a saturated state, were 1.18, 1.19, and 1.24 times higher as compared with the completely dry state, respectively. The elasticity modulus of the concrete with high-strength grade was more sensitive to the water saturation, under the same water saturation condition.

## Figures and Tables

**Table 1:** Mix proportion and the main parameter of concrete.

Strength Grade	Water/Cement Ratio	Mix Proportion/kg·m^−3^				
		Water	Cement	Medium Sand	Small Stone	Middle Stone
C15	0.65	158	243	729	709	709
C20	0.55	150	273	615	685	685
C30	0.42	165	391	581	646	646

**Table 2:** Test grouping table.

Strength Grade	Completely Dry Group	Soaking Time/h				
		3	10	24	72	240
C15	A_0_	A_1_	A_2_	A_3_	A_4_	A_5_
C20	B_0_	B_1_	B_2_	B_3_	B_4_	B_5_
C30	C_0_	C_1_	C_2_	C_3_	C_4_	C_5_

**Table 3:** Axial compressive strength of concrete under different water saturation values.

C15		C20		C30	
Water Saturation/%	Axial Compressive Strength/MPa	Water Saturation/%	Axial Compressive Strength/MPa	Water Saturation/%	Axial Compressive Strength/MPa
0.00	16.41	0.00	21.48	0.00	28.29
47.56	14.87	71.63	19.85	47.29	26.67
78.30	13.30	80.48	18.55	78.05	24.45
97.99	12.45	80.90	17.75	81.51	23.50
99.59	12.07	92.75	17.33	90.48	23.31
100	11.94	100	16.94	100	22.42

**Table 4:** Elasticity modulus of concrete under different water saturation values.

C15		C20		C30	
Water Saturation/%	Elastic Modulus/GPa	Water Saturation/%	Elastic Modulus/GPa	Water Saturation/%	Elastic Modulus/GPa
0	19.58	0	22.50	0	24.31
47.56	20.83	71.63	24.58	47.29	27.12
78.30	22.94	80.48	25.19	78.05	27.23
97.99	23.24	80.90	24.75	81.51	28.03
99.59	23.04	92.75	26.08	90.48	29.26
100	23.24	100	26.63	100	30.36

**Table 5:** Curve fitting parameters of prediction Equations (4).

Strength Grade	Model Parameter		
	α	β	$R^{2}$
C15	0.038	19.45	0.96
C20	0.037	22.27	0.90
C30	0.052	24.27	0.89

**Table 6:** Predicted value by Equation (4) and tested value reference [19].

Water Saturation Si/%	Predicted Value by Equation (4) Elasticity Modulus/GPa	Tested Value Reference [19] Elasticity Modulus/GPa	Relative Error/%
0.00	24.27	25.01	−2.97
35.14	26.10	27.59	−5.42
75.68	28.21	29.82	−5.42
72.97	28.06	29.80	−5.81
100.00	29.47	32.47	−9.23

Note: The relative error is the ratio of difference between measured value and predicted value to measured value.
